# Genetic Characterization of Intimin Gene (*eae*) in Clinical Shiga Toxin-Producing *Escherichia coli* Strains from Pediatric Patients in Finland

**DOI:** 10.3390/toxins15120669

**Published:** 2023-11-23

**Authors:** Lei Wang, Xiangning Bai, Elisa Ylinen, Ji Zhang, Harri Saxén, Andreas Matussek

**Affiliations:** 1 Department of Microbiology, Division of Laboratory Medicine, Oslo University Hospital and University of Oslo, 0372 Oslo, Norway; lei.wang@medisin.uio.no (L.W.); xiangning.bai@medisin.uio.no (X.B.); 2 Jinan Center for Disease Control and Prevention, Jinan 250021, China; 3 Department of Clinical Microbiology, Division of Laboratory Medicine, Karolinska Institutet, 141 52 Stockholm, Sweden; 4 Department of Pediatric Nephrology and Transplantation, New Children’s Hospital, University of Helsinki and Helsinki University Hospital, 00029 Helsinki, Finland; elisa.ylinen@hus.fi (E.Y.); harri.saxen@hus.fi (H.S.); 5 Fonterra Research and Development Centre, Dairy Farm Road, Palmerston North 4442, New Zealand; ji.zhang@fonterra.com

**Keywords:** Shiga toxin (Stx)-producing *Escherichia coli*, intimin, *eae* subtype, hemolytic uremic syndrome, pediatric patients

## Abstract

Shiga toxin (Stx)-producing *Escherichia coli* (STEC) infections cause outbreaks of severe disease in children ranging from bloody diarrhea to hemolytic uremic syndrome (HUS). The adherent factor intimin, encoded by *eae*, can facilitate the colonization process of strains and is frequently associated with severe disease. The purpose of this study was to examine and analyze the prevalence and polymorphisms of *eae* in clinical STEC strains from pediatric patients under 17 years old with and without HUS, and to assess the pathogenic risk of different *eae* subtypes. We studied 240 STEC strains isolated from pediatric patients in Finland with whole genome sequencing. The gene *eae* was present in 209 (87.1%) strains, among which 49 (23.4%) were from patients with HUS, and 160 (76.6%) were from patients without HUS. O157:H7 (126, 60.3%) was the most predominant serotype among *eae*-positive STEC strains. Twenty-three different *eae* genotypes were identified, which were categorized into five *eae* subtypes, i.e., γ1, β3, ε1, θ and ζ3. The subtype *eae*-γ1 was significantly overrepresented in strains from patients aged 5–17 years, while β3 and ε1 were more commonly found in strains from patients under 5 years. All O157:H7 strains carried *eae*-γ1; among non-O157 strains, strains of each serotype harbored one *eae* subtype. No association was observed between the presence of *eae*/its subtypes and HUS. However, the combination of *eae*-γ1+*stx2a* was significantly associated with HUS. In conclusion, this study demonstrated a high occurrence and genetic variety of *eae* in clinical STEC from pediatric patients under 17 years old in Finland, and that *eae* is not essential for STEC-associated HUS. However, the combination of certain *eae* subtypes with *stx* subtypes, i.e., *eae*-γ1+*stx2a*, may be used as risk predictors for the development of severe disease in children.

## 1. Introduction

Shiga toxin (Stx)-producing *Escherichia coli* (STEC) is a major cause of human gastrointestinal diseases ranging from mild, watery to bloody diarrhea. The severe complications associated with toxin production and release can cause the life-threatening hemolytic uremic syndrome (HUS), leading to kidney failure and neurological episodes [1]. It has been reported that 6–25% of patients infected with STEC develop HUS, and the rate is higher in children [2,3,4]. More than 1000 different serotypes of STEC have been defined in humans, animals and the environment [5,6,7]. O157:H7 is the predominant serotype causing more severe disease. However, non-O157 serogroups such as O26, O45, O103, O111, O121, and O145 (referred to as the ‘top six’ non-O157 STEC) have been increasingly reported in outbreaks and sporadic cases in recent years in different countries like Europe and North America [8,9,10].

Stx1 and Stx2 are the main virulence factors of STEC. There are at least three *stx1* subtypes (*stx1a*, *stx1c*, and *stx1d*) and twelve *stx2* subtypes (*stx2a-stx2l)*, associated with different clinical outcomes and disease severity [11,12,13,14,15]. *stx2a*, *stx2c*, and *stx2d* subtypes are frequently related to a higher risk of HUS, whereas other subtypes such as *stx2e*, *stx2b*, *stx2f*, and *stx2g* are related to less severe illnesses [11,16,17]. Besides Stx, intimin encoded by *eae* is considered as an important virulence factor in STEC. *eae* resides on the locus of enterocyte effacement (LEE) pathogenicity island and implicates attaching and effacing lesions in intestinal cells, attributing to determining the course of STEC infections [18]. Based on the difference of intimin C-terminal where cellular binding activity is highly variable, more than 30 intimin subtypes have been identified [19], and the most common subtypes are α, β, γ, ε, ζ, and η [20]. Different *eae* subtypes are associated with host specificity and tissue sensitivity. *eae*-β has been shown to predominate in non-O157 STEC strains from diarrheal patients, and *eae*-γ1 is associated with severe symptoms [21]. *eae*-ζ tends to be present in isolates from cattle [22,23]. *eae* subtypes are often associated with particular serotypes or pathotypes [24]. The serotypes O157:H7 and O145:H28 are linked to the *eae*-γ1, whereas O26:H11, O103:H2, and O111:H8 tend to carry *eae*-β1, *eae*-ε, and *eae*-θ, respectively [25]. *stx*2 and *eae* are commonly found in STEC strains linked with severe clinical outcomes [26]. 

Studies on the molecular characteristics of *eae* in clinical STEC strains are limited, especially in pediatric patients, and the relationship between *eae* subtypes and clinical symptoms remains to be addressed. In this study, we investigated the subtypes and polymorphisms of *eae* among clinical STEC strains from pediatric patients under 17 years old in Finland, and assessed the association of *eae* subtypes with clinical symptoms (HUS and non-HUS).

## 2. Results

### 2.1. Prevalence of eae in STEC Isolates

Among 240 STEC isolates, *eae* was present in 209 (87.1%) strains, including 49 (94.2%) strains from HUS patients, and 160 (85.1%) from non-HUS patients. All O157:H7 strains (n = 126) and 83 out of 114 (72.8%) non-O157 strains harbored *eae* (Table 1). *eae* is significantly associated with O157:H7 strains (*p* < 0.0001) (Table 1). No association was observed between the presence of *eae* and HUS status, age, or sex (Table 1).

### 2.2. Diversity of eae

**Figure 1:**
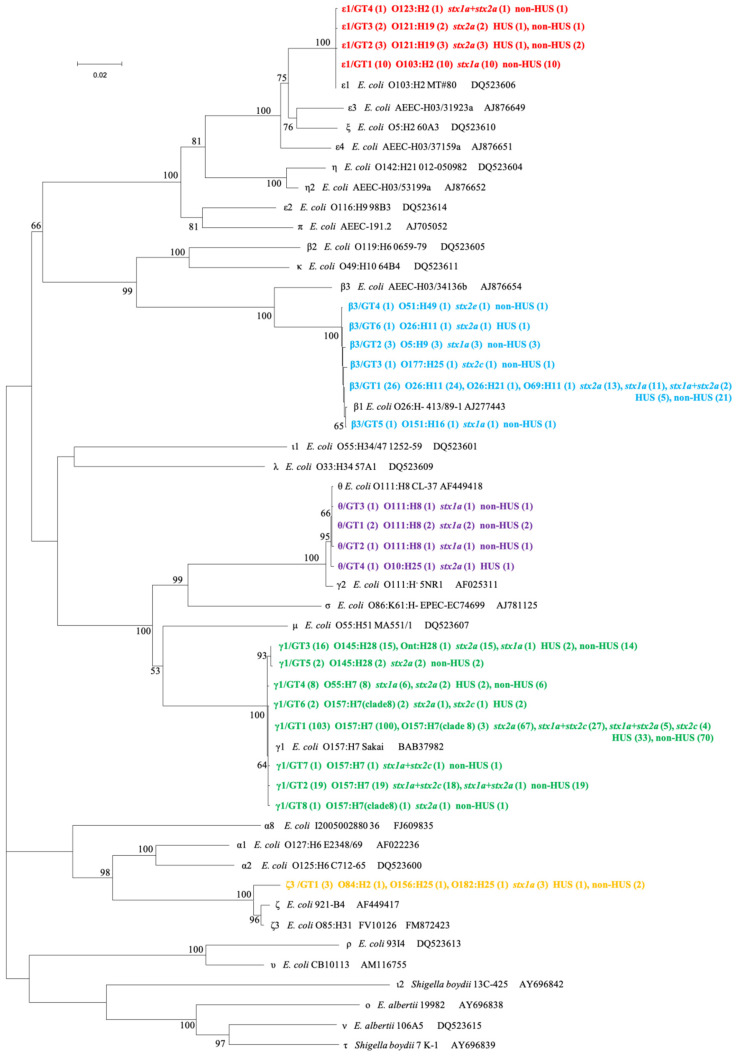
Phylogenetic relationships of 23 unique *eae* sequences identified in this study (indicated in bold and different colors) and 30 reference sequences representing different *eae* subtypes based on the neighbor-joining method. For the branches that represent the identified 23 *eae* subtypes in this study, the tips are labeled in the order of *eae* subtype/genotype, serotype, *stx* subtype, and HUS status. The number of the strains are shown in brackets. Scale bar indicates genetic distance.

In total, 209 complete *eae* sequences were extracted from all *eae*-positive STEC genomes, among which 23 unique sequences were identified, with the nucleotide identities ranging from 90.45% to 99.98%. The 23 sequences were assigned into five *eae* subtypes, namely ε1, γ1, β3, θ, and ζ3 (Figure 1). *eae*-γ1 was present in 152 (72.7%) strains, being the most predominant subtype, followed by β3 (33, 15.8%) and ε1 (16, 7.7%). *eae* sequence polymorphism was further illustrated by genotypes (GTs) within a subtype. γ1 subtype showed the highest diversity with eight GTs (GT1-GT8), followed by β3 (GT1-GT6), ε1 (GT1-GT4), θ (GT1-GT4), and ζ3 (GT1) (Figure 1). Using BLASTn search against the GenBank database (nr/nt), 13 *eae* genotypes showed 100% nucleotide identity to the publicly available *eae* sequences, while 10 *eae* genotypes (γ1/GT2, γ1/GT7, γ1/GT8, β3/GT3, β3/GT4, β3/GT5, β3/GT6, ε1/GT3, θ/GT2, and θ/GT4) showed 99.89–99.96% nucleotide identity to the publicly available sequences in the database (accessed 17/8/2023). Notably, γ1 was statistically significantly overrepresented in strains from patients aged 5 to 17 years (*p* < 0.0001), while β3 and ε1 were significantly higher in strains from patients under 5 years (*p* = 0.001 and *p* = 0.037) (Table 2). No statistically significant difference was found between different *eae* subtypes and HUS status.

### 2.3. Association of eae Subtypes/Genotypes with Serotypes

Nineteen serotypes were identified among 209 *eae*-positive STEC strains. O157:H7 was the most predominant serotype (126 strains, 60.3%), followed by O26:H11 (25, 12.0%), O145:H28 (17, 8.1%), O103:H2 (10, 4.8%), O55:H7 (8, 3.8%), O121:H19 (5, 2.4%), O111:H8 (4, 1.9%), and O5:H9 (3, 1.4%). All O157:H7 strains carried *eae*-γ1; among non-O157 strains, strains of each serotype harbored one *eae* subtype (Table S2). Strains of serotype O145:H28 and O55:H7 carried *eae*-γ1; O26:H11, O26:H21, O5:H9, O51:H49, O69:H11, O177:H25, and O151:H16 strains carried *eae*-β3; O103:H2, O121:H19, and O123:H2 strains carried *eae*-ε1; O111:H8 and O10:H25 strains carried *eae*-θ; and O156:H25, O182:H25, and O84:H2 strains carried *eae*-ζ3 (Table S2). *eae*-γ1 was statistically significantly overrepresented in O157:H7 strains (*p* < 0.0001), while *eae*-ε1, *eae*-β3, and *eae*-θ were more prevalent in non-O157 strains (*p* < 0.0001) (Table 2). Among the six clade 8 O157:H7 strains, two strains from patients with HUS harbored *eae*-γ1/GT6 (Figure 1).

### 2.4. Characterization of stx Subtypes in eae-Positive STEC Isolates in Relation to HUS

One *stx1* subtype (*stx1a*) and three *stx2* subtypes (*stx2a*, *stx2c,* and *stx2e*) were identified in 209 *eae*-positive STEC strains. Six *stx* subtypes combinations were present, including *stx2a* (108 strains), *stx1a+stx2c* (46), *stx1a* (39), *stx1a+stx2a* (9), *stx2c* (6), and *stx2e* (1). *stx2* (115, 55.0%) was the most prevalent, especially in strains from HUS patients (46, 93.9%), and *stx2a* (108, 51.7%) was the most predominant *stx* subtype (Table 3 and Table S3). An association was observed between *stx* subtypes and *eae* subtypes. All strains carrying *stx1a+stx2c* harbored *eae*-γ1, and *eae*-γ1 was also overrepresented in strains carrying *stx2a* (*p* = 0.0049). *eae*-γ1 was less prevalent in strains with *stx1a* (*p* < 0.0001), while *eae*-β3 was less prevalent in strains with *stx1a+stx2c* (*p* = 0.0002). *eae*-β3, *eae*-ε1, *eae*-θ, and *eae*-ζ3 were more prevalent in strains with *stx1a* (*p* < 0.0001, *p* < 0.0001, *p* = 0.0046, and *p* = 0.0061, respectively) (Table 2 and Table S3).

An association between *stx* subtypes and HUS status or age was analyzed in the presence of *eae*. *stx1a*+*stx2c*+*eae*-γ1 and *stx1a*+*eae*-β3 were more prevalent in non-HUS-associated strains (*p* < 0.0001and *p* = 0.0245), while *stx2a*+*eae*-γ1 was significantly associated with HUS (*p* < 0.0001). In addition, *stx1a+stx2c*+*eae*-γ1 was more prevalent in strains from patients aged 5–17 than in those under 5 years (*p* = 0.0071) (Table 3).

Additionally, the relationship between a combination of *stx*+*eae*+serotypes (O157 and non-O157) and HUS status was evaluated. *stx2a*+*eae*-γ1+O157:H7 was strongly associated with HUS, while *stx1a*+*eae*-β3+non-O157 and *stx1a*+*stx2c*+*eae*-γ1+O157:H7 were associated with non-HUS (Table S4).

### 2.5. Comparison with eae-Positive STEC Strains from Swedish Pediatric Patients

In a previous study in Sweden [21], *eae*-γ1 was found to be statistically overrepresented in clinical *eae*-positive STEC strains from HUS patients, while β3 was significantly higher in non-HUS STEC strains from both adults and children. In addition, *eae* was found to be more prevalent in strains from children; however, the relationship between *eae* subtypes and clinical outcomes in strains from children was unclear in Sweden. We were interested to know if the correlation between *eae* subtype and clinical outcome was age-dependent. We then extracted 110 *eae*-positive strains from Swedish patients under 17 years old, and performed the same statistical analysis on the Swedish strains, as well as all 319 strains, including 110 from Swedish patients and 209 from Finnish patients within the same age group (under 17 years). The results showed that the presence of *eae* and the *eae*-γ1 subtype was significantly higher in strains from patients with HUS compared to strains from patients without HUS under 17 years old in Sweden (*p* = 0.0005 and *p* < 0.0001) (Table 4). The same correlation was observed in merged strains from Sweden and Finland under 17 years old (Table S5). *eae*-β3 was significantly higher in strains from non-HUS-STEC patients in Sweden, and in merged non-HUS-STEC strains from the two countries compared to HUS-STEC strains (*p* = 0.0001 and *p* = 0.0033) (Table 4 and Table S5).

## 3. Discussion

Over 90% of pediatric HUS cases are caused by gastrointestinal infection with STEC [2,27,28]. The reasons why children are more commonly affected than adults are unknown, but potential explanations may relate to the ability of specific strains to establish disease in specific populations, the age-specific expression on cells of receptors for toxins, or diameters of renal blood vessels. The presence of *eae* has been reported to be significantly associated with disease severity, and has previously been identified as a risk factor for the development of HUS. However, information about the characteristics and genetic diversity of *eae* and their contribution and correlation to disease severity is currently limited, especially in pediatric patients. In this study, *eae* was present in 87.1% of clinical pediatric strains, in accordance with previous studies, revealing that most clinical STEC strains possess *eae* [29,30]. The majority of *eae*-positive strains were of serotype O157:H7 (60.3%). It is worth noting that 72.8% of non-O157 strains in this study were *eae* positive, which was higher than previous findings reported in England (52.5%) [31] and Sweden (62.1%) [21]. However, the prevalence was reported in strains from children and adult patients together. This may indicate that *eae* is more prevalent in STEC strains from children than adults. The difference may be also due to the variations between geographical regions, sex, etc. 

Various *eae* subtypes may confer distinct colonization patterns within the human intestine, thus leading to distinct pathogenic capability. STEC strains with *eae*-β, ε, γ1, θ, and ζ subtypes have been reported to be more virulent, and thus pose a higher pathogenic risk [21,25]. Previously, *eae*-γ1-positive STEC strains were isolated from children with HUS in Uruguay, highlighting the clinical significance of *eae*-γ1 [32]. In this study, five *eae* subtypes were identified (*eae*-γ1, β3, ε1, θ, and ζ3), of which *eae*-γ1 was the most prevalent, followed by β3 and ε1. In addition, *eae*-γ1 was more prevalent in children aged 5–17 years compared to children below 5 years, while β3 and ε1 were more prevalent in strains from children under 5 years, indicating that various *eae* subtypes tended to be present in different age groups. *eae*-γ1 was significantly associated with O157:H7 strains. Consistent with the previous findings, we found that *eae*-γ1 was carried by serogroup O157 and O145 strains; *eae*-β3 was carried by O26 strains; *eae*-ε1 was carried by O103 and O121 strains; and *eae*-θ was carried by O111 strains [25]. Certain *eae* subtypes may play roles in the pathogenicity of these clinical high-risk strains, leading to different clinical manifestations. No association was found between the presence of *eae*/its subtypes and HUS status in Finland. Interestingly, the presence of *eae* and *eae*-γ1 was significantly associated with HUS in patients within the same age in Sweden.

STEC strains producing Stx2a or Stx2c subtypes are more associated with HUS in humans, especially with the presence of *eae* [33]. We found that the presence of *stx2a*+*eae*-γ1 was strongly associated with HUS, while *stx1a*+*eae*-β3 and *stx1a+stx2c*+*eae*-γ1 were associated with non-HUS. This supports that the combination of *stx2a*+*eae* (particularly *eae*-γ1) can be used in risk assessments for the development of HUS and severe disease outcomes.

To conclude, our study showed high prevalence (87.1%) and genetic diversity of *eae* in clinical pediatric STEC strains. No correlation was observed between the presence of the *eae* gene/its subtypes and HUS status in pediatric STEC strains in Finland, while the combination of *stx2a+eae*-γ1 was significantly higher in STEC strains from patients with HUS.

## 4. Materials and Methods

### 4.1. Collection of eae-Positive STEC Isolates and Metadata

STEC strains were collected from STEC-infected pediatric patients under 17 years old in Finland between 2000 and 2016, and the clinical information was retrieved from the medical records as previously described [34]. Patients were classified into HUS and non-HUS groups according to clinical manifestation. Whole genome sequencing (WGS), assembly, and annotation were carried out as previously reported [35]. Genetic characterization of STEC strains, including *stx* subtyping, determination of serotypes, the presence of intimin encoding gene *eae,* and the clade-8 specific SNP were carried out as described previously [35]. Metadata of all STEC isolates, including strain ID, serotype, clade 8, presence of *eae* gene, *eae* subtype and genotype, *stx* subtype, and accession numbers of genomes, as well as clinical information of patients, including HUS status, age, and sex, are listed in Supplementary Table S1.

To examine the relationship between *eae* subtypes and HUS status in pediatric patients in different countries, STEC strains from STEC-infected patients under 17 years old in Sweden reported previously [21] were extracted and analyzed. 

### 4.2. eae Subtyping

According to the genome annotation, complete *eae* sequences were extracted from the genome assemblies of 209 *eae*-positive STEC isolates, and then aligned with reference nucleotide sequences of *eae* subtypes downloaded from GeneBank. MEGA 11 software (Center for Evolutionary Medicine and Informatics, Tempe, AZ, USA) was used to compute the genetic distances of the *eae* subtypes with the maximum composite likelihood method, and a neighbor-joining phylogenetic tree was generated using 1000 bootstrap resampling. The phylogenetic tree structure and genetic distance were employed to determine the *eae* subtype. The diversity within each *eae* subtype was determined by *eae* GTs based on *eae* sequence polymorphism, as previously described [23]. 

### 4.3. Statistical Analyses

A statistical association between the existence of *eae*/its subtypes and strain characteristics (serotypes, *stx* subtypes) or clinical outcomes (HUS and non-HUS) was assessed with Fisher’s exact test using R software version 4.3.1 (https://www.r-project.org) (accessed on 20 August 2023), and a *p*-value below 0.05 was considered statistically significant. 

## Figures and Tables

**Table 1:** Prevalence of *eae* in 240 STEC strains isolated from pediatric patients (<17 years) ^#^.

*eae*	Serotype		*p*-Value	Clinical Symptom		*p*-Value	Age Group		*p*-Value	Sex		*p*-Value
	O157:H7 (n = 126)	Non-O157 (n = 114)		HUS (n = 52)	Non-HUS (n = 188)		<5 Years (n = 117)	5–17 Years (n = 123)		Male (n = 122)	Female (n = 118)	
Positive	126 (100.0)	83 (72.81)	<0.0001 *	49 (94.23)	160 (85.13)	0.1025	102 (87.18)	107 (86.99)	1	103 (84.43)	106 (89.83)	0.2502

^#^ The association was analyzed between *eae* gene and serotypes (O157 and non-O157), clinical symptoms (HUS and non-HUS), age groups (<5 years and 5–17 years; the age was grouped according to the median age of 5 years), sex (male and female). The number represents the number of *eae*-positive strains, and percentage was shown in brackets. * Statistically significant difference.

**Table 2:** Association between *eae* subtypes and serotypes or age or *stx* subtypes ^#^.

*eae* Subtype (No. of Strains)	Serotype					Age Group					*stx* Subtype								
	O157:H7 (n = 126)		Non-O157 (n = 83)		*p*-Value	<5 Years (n = 102)		5–17 Years (n = 107)		*p*-Value	*stx2a* (n = 108)		*p*-Value	*stx1a* (n = 39)		*p*-Value	*stx1a*+*stx2c* (n = 46)		*p*-Value
	Pos	Prevalence	Pos	Prevalence		Pos	Prevalence	Pos	Prevalence		Pos	Prevalence		Pos	Prevalence		Pos	Prevalence	
γ1 (152)	126	100.0%	26	31.33%	<0.0001 *	60	58.82%	92	85.98%	<0.0001 *	88	81.48%	0.0049 *	7	17.95%	<0.0001 *	46	100.00%	<0.0001 *
β3 (33)	0	0.00%	33	39.76%	<0.0001 *	25	24.51%	8	7.48%	0.001 *	14	12.96%	0.261	15	38.46%	<0.0001 *	0	0.00%	0.0002 *
ε1 (16)	0	0.00%	16	19.28%	<0.0001 *	12	11.76%	4	3.74%	0.037 *	5	4.63%	0.1187	10	25.64%	<0.0001 *	0	0.00%	0.0255
θ (5)	0	0.00%	5	6.02%	0.0092 *	3	2.94%	2	1.87%	0.6772	1	0.93%	0.200	4	10.26%	0.0046 *	0	0.00%	0.5883
ζ3 (3)	0	0.00%	3	3.61%	0.0613	2	1.96%	1	0.93%	0.6143	0	0.00%	0.1111	3	7.69%	0.0061 *	0	0.00%	1

^#^ The association was analyzed between *eae* subtype and clinical symptoms (HUS and non-HUS), age groups (<5 years and 5–17 years; the age was grouped according to the median age of 5 years), serotypes (O157 and non-O157), sex (male and female), as well as *stx* subtypes (*stx2a*, *stx1a*, *stx2c*, *stx2e*, *stx1a*+*stx2a*, and *stx1a*+*stx2c*), only variables with statistically significant differences (*p* < 0.05) were shown in this table. * Statistically significant difference. pos: number of positive strains.

**Table 3:** Association between *stx*+*eae* and HUS status or age or serotypes ^#^.

*stx+eae*	No. of Strains	Clinical Symptom		*p*-Value	Age Group		*p*-Value	Serotype		*p*-Value
		HUS (n = 49)	Non-HUS (n = 160)		<5 Years (n = 102)	5–17 Years (n = 107)		O157:H7 (n = 126)	Non-O157 (n = 83)	
***stx1*** **+** ***eae***	39	1 (2.04)	38 (23.75)	0.0002 *	24 (23.53)	15 (14.02)	0.1092	0 (0.00)	39 (46.99)	<0.0001 *
*stx1a*+*eae*-β3	15	0 (0.00)	15 (9.38)	0.0245 *	-	-	-	0 (0.00)	15 (18.07)	<0.0001 *
*stx1a*+*eae*-ε1	10	0 (0.00)	10 (6.25)	0.1212	-	-	-	0 (0.00)	10 (12.05)	<0.0001 *
*stx1a*+*eae*-γ1	7	0 (0.00)	7 (4.37)	0.2033	-	-	-	0 (0.00)	7 (8.43)	0.0013 *
*stx1a*+*eae*-θ	4	0 (0.00)	4 (2.50)	0.5750	-	-	-	0 (0.00)	4 (4.82)	0.0267 *
*stx1a*+*eae*-ζ3	3	1 (2.04)	2 (1.25)	0.5739	-	-	-	0 (0.00)	3 (3.61)	0.0667
***stx2*** **+** ***eae***	115	46 (93.88)	69 (43.13)	<0.0001 *	61 (59.80)	54 (50.47)	0.2108	74 (58.73)	41 (49.40)	0.2028
*stx2e*+*eae*-β3	1	0 (0.00)	1 (0.63)	1	1 (0.98)	0 (0.00)	0.4880	0 (0.00)	1 (1.20)	0.3971
*stx2c*+*eae*-γ1	6	2 (4.08)	4 (2.50)	0.6267	2 (1.96)	4 (3.74)	0.6835	5 (3.97)	1 (1.20)	0.4059
*stx2a*+*eae*-γ1	88	35 (71.43)	53 (33.13)	<0.0001 *	-	-	-	-	-	-
*stx2a*+*eae*-β3	14	6 (12.24)	8 (5.00)	0.0997	-	-	-	-	-	-
*stx2a*+*eae*-ε1	5	2 (4.08)	3 (1.89)	0.3341	-	-	-	-	-	-
*stx2a*+*eae*-θ	1	0 (0.00)	1 (0.63)	1	-	-	-	-	-	-
***stx1*** **+** ***stx2*** **+** ***eae***	55	2 (4.08)	53 (33.13)	<0.0001 *	17 (16.67)	38 (35.51)	0.0027 *	52 (41.27)	3 (3.61)	<0.0001 *
*stx1a*+*stx2a* +*eae*-β3/γ1	9	2 (4.08)	7 (4.38)	1	3 (2.94)	6 (5.61)	0.4993	6 (4.76)	3 (3.61)	1
*stx1a*+*stx2c* +*eae*-γ1	46	0 (0.00)	46 (28.75)	<0.0001 *	14 (13.73)	32 (29.91)	0.0071 *	46 (36.51)	0 (0.00)	<0.0001 *

^#^ The association was analyzed between *eae*+*stx* subtype and clinical symptoms (HUS and non-HUS), age groups, and serotypes. The number represents the number of strains carrying specific *stx*+*eae* subtypes, and percentage is shown in brackes. * Statistically significant difference. pos: number of positive strains.

**Table 4:** Association between *eae* subtypes and HUS status in pediatric patients under 17 years old in Sweden ^#^.

*eae* Subtype	No. of Strains	HUS (34)		Non-HUS (76)		*p*-Value
		Pos	Prevalence	Pos	Prevalence	
γ1	40	22	64.71%	18	23.68%	<0.0001 *
ε1	35	9	26.47%	26	34.21%	0.5091
β3	28	1	2.94%	27	35.53%	0.0001 *
θ	5	2	5.88%	3	3.95%	0.6436
ζ3	1	0	0.00%	1	1.32%	1
ρ	1	0	0.00%	1	1.32%	1
In total	110	34	97.14%	76	70.37%	0.0005 *

^#^ The association was analyzed between *eae*/its subtype and clinical symptoms (HUS and non-HUS) in strains from pediatric patients in Sweden. * Statistically significant difference. pos: number of positive strains.

## Data Availability

The assemblies of all strains in this study were deposited in GenBank with accession numbers and metadata shown in Table S1.

## Supplementary Materials

The following supporting information can be downloaded at: https://www.mdpi.com/article/10.3390/toxins15120669/s1, Table S1: Metadata of 240 clinical STEC isolates in this study; Table S2: Association between *eae* subtypes and serotypes; Table S3: Association between *eae* subtypes and *stx* subtypes; Table S4: Association between combination of *stx*+*eae*+serotypes (O157/non-O157) and HUS status; Table S5: Association between *eae*/its subtypes and HUS status in strains from pediatric patients (<17 years) in Sweden and Finland, respectively, and in merged strains from the two countries.
